# HOSPITALIZATION AT HOME MAY ADDRESS SOCIAL DETERMINANTS OF HEALTH: RESULTS FROM A QUALITATIVE STUDY

**DOI:** 10.1093/geroni/igac059.2420

**Published:** 2022-12-20

**Authors:** Gabrielle Schiller, Heather Wurtz, Sybil Masse, Abigail Baim-Lance, Alex Federman, Emily Franzosa, Ksenia Gorbenko, Linda DeCherrie

**Affiliations:** CUNY School of Public Health, Astoria, New York, United States; Icahn School of Medicine at Mount Sinai, New York, New York, United States; Icahn School of Medicine at Mount Sinai, New York, New York, United States; Icahn School of Medicine at Mount Sinai, New York, New York, United States; Icahn School of Medicine at Mount Sinai, New York, New York, United States; Icahn School of Medicine at Mount Sinai, New York, New York, United States; Icahn School of Medicine at Mount Sinai, New York, New York, United States; Medically Home, Boston, Massachusetts, United States

## Abstract

This project evaluated implementation of the Acute Hospital Care at Home (AHCaH) waiver from the U.S. Center for Medicare & Medicaid Services (CMS), launched in November 2020 to ensure availability of acute care as COVID-19 cases overwhelmed hospitals. We interviewed 18 HaH program leaders of 14 programs, from both existing and newly launched HaH programs, about their experiences applying for and implementing HaH under the CMS waiver. A thematic analysis of the interviews was completed. Informants described HaH programs as an opportunity for healthcare professionals to have “eyes in the home,” referring to ways in which the HaH team was able to observe and intervene in aspects of the patient’s life not accessible from traditional in-patient care. Informants described various ways that HaH programs addressed Social Determinants of Health (SDoH) including assessing patients’ ability to obtain healthy foods, reducing potential safety hazards in the home, and providing education and support to patients’ caregivers. Informants also suggested that HaH may reduce anxiety associated with hospital stay and separation from loved ones, helping people already contending with social inequities. Addressing SDoH is important for ensuring quality care and long-term positive changes in the patient’s lifestyle and environment. As CMS shifts their aims to further address the SDoH, our findings suggest that the interdisciplinary care approach of HaH programs may be a model of addressing both social and medical needs.

